# Analysis of an ‘Official Report to the Surgeon-General U. S. Army, on the Use of Large Doses of Sulphate of Quinine in Diseases of the South

**Published:** 1845-09

**Authors:** John Byrne

**Affiliations:** Assistant Surgeon U. S. Army; Surgeon-General’s Office


*Analysis of an "Official Report to the Surgeon-General U. S. Army, on the use of Large Doses of Sulphate of Quinine in Diseases of the South.* By JOHN BYRNE, M.D., Assistant Surgeon U. S. Army."

Dr. Byrne's experience in the use of large doses of quinine was acquired during a three years' tour of duty in Florida, at a period when the greatest number of troops were operating in the Territory against the Indians, and when diseases of miasmatic origin prevailed very generally and with great severity. During the early part of his service, Dr. B. was stationed at comparatively healthy posts, where fever cases were neither very frequent nor malignant, and the treatment of intermittents and remittents was very similar to that ordinarily pursued at the North, and it was attended by the usual success. At a later period, however, he was in charge of large bodies of men, occupying positions characterized by extreme unhealthiness, and for the last two years of his Florida service his reports of sick range from 15 to 200 daily, exclusive of camp followers, families and citizens.

In individuals who had been but for a short time exposed to the climate of Florida, and in recruits recently arrived from the North, it was remarked that fevers were attended by high arterial action and much disposition to local inflammation; whilst in the older residents of the country, cases of disease were uniformly marked by debility and loss of vital force. In the former, antiphlogistics, diaphoretics, and other preparatory measures, were generally required before the administration of quinine in any form. The latter, on the contrary—by far the greater number—were rarely accompanied by those evidences of diseased secretions so much dilated upon by authors; the tongue was often perfectly clean, and the alvine discharges natural in character and frequency. Here quinine was given at once, and in decided doses; the use of preparatory measures served but to weaken the patient and to protract his recovery.

Imbued with the idea inculcated at the North, that quinine is to be used in intermittents, only after a careful *preparation of the system*, and that in remittents its administration is rarely justifiable under any circumstances, Dr. B. expresses his regret that, in the earlier periods of his southern service, he frequently permitted patients to be worn down with repeated paroxysms of fever rather than have recourse to quinine—because no decided chill or lengthened intermission marked the case as one of intermittent fever. Subsequently he learned the value of quinine in all forms of malarial fever, and had the satisfaction of being able to arrest at once the chain of morbid symptoms, which under the old system of treatment were left to *wear themselves out* by repetition or to terminate in death.

In intermittents, and remittents of ordinary severity, it is considered as a matter of indifference whether the quinine is given in large or small doses—provided that 10 to 20 grains of the medicine are introduced into the system within a certain number of hours before the expected paroxysm. By a series of experiments instituted with the view of testing the comparative influence of the large and small doses (20 grains and 2 grains) in producing disturbance of the head, it was determined that in this respect, no difference existed between them, and that the liability to cerebral symptoms depended rather upon the condition of the nervous system, than upon the number of grains administered.

In the severe forms of remittent, however, and particularly in congestive fever, the size of the dose to be given is no longer a matter of choice; here there is no time to introduce the medicine gradually into the system, and if there were time, it is doubtful whether the small doses would produce a sufficiently decided impression upon the disease. It is in *congestive fever* that the value of decided doses of quinine is most strongly marked.

A man, previously in good health, is suddenly stricken down—overpowered by miasmatic poison; he appears insensible—his countenance presents a vacant stare, and he is aroused with difficulty; when questioned as to his condition he insists that there is nothing the matter with him, and begs to be left undisturbed. His extremities are cold, skin elsewhere generally not above the natural temperature, and sometimes covered with clammy moisture; his breathing is laborious. In accordance with ordinarily received opinions, these symptoms would be ascribed to *congestion* of the internal organs, and free *venesection* the remedy applied. A physician familiar with the use of quinine in Florida, would think and act differently. He would view the case as one of poisoning by malaria, and the congestion—not as the first link in the chain of morbid action—but the mere consequence of the poisonous impression upon the nervous centres. Experience has taught him that the antidote to this poisonous impression is quinine; and accordingly he would order a scruple of the remedy to be given at once, combined with capsicum, laudanum, turpentine or camphor, to be repeated as often as necessary—with warmth, and stimulating frictions to the surface. Under this treatment re-action is established in the course of a few hours, and but little else is required to insure the safety of the patient—the occurrence of another paroxysm being prevented by the amount of quinine already in the system.

What would have been the result in this case if the lancet had been used, and the immediate and decided administration of quinine neglected?

Cases of this kind were not of rare occurrence in Florida, and where quinine was used with a sparing hand they were not unfrequently fatal in a few hours.

Dr. B. rejects entirely the idea that periodical fevers are checked with more ease and certainty on the critical days. At first crises were looked for, and remedies administered in anticipation of their occurrence, without success; but later, when the full powers of quinine were learned, it was considered surer practice, and certainly a more effective one in military service, to cut short the disease at once; and thus the danger was avoided of allowing a quotidian intermittent to run into the remittent form, which in the expectant mode of treatment was often the case. No bad consequences followed this mode of practice, nor in any case were injurious effects perceived from the large doses of quinine administered, and it is asserted that no case of intermittent was encountered in Florida in which, when properly administered, the sulphate of quinine failed to arrest the disease; its permanent cure, however, was only to be effected when the patient was removed from contact with the miasmatic poison. No cases occurred in which the bark, in substance, was found more efficacious than its active principle.

In numerous instances the administration of quinine in moderate doses, combined with calomel and Dover's powder, in chronic dysentery, particularly when this disease was accompanied by fever, was followed by the happiest effects. This circumstance, in connection with the fact that diarrhœa and dysentery invaribly prevailed simultaneously with the ordinary forms of miasmatic fever, goes far to confirm the belief in their common origin, particularly in malarial districts. That the habitual free use of quinine increases the tendency to disease of the bowels, of the liver, or spleen, is most strongly doubted; and it is considered that this notion may fairly take its place amongst the obsolete prejudices of times gone by.

In frequent *post-mortem* examinations of cases of chronic dysentery, with repeated attacks of fever—the form of disease which produces most of the deaths in Florida—Dr. B. expresses his surprise that organic alterations of the liver were so rarely encountered. "From the fact that quinine," he remarks, "was so largely used in the Territory, and that I rarely met with diseased livers, enlarged spleens, or dropsy as a consequence of fever, I must acquit this drug of the charge often brought against it of inducing these affections. When they do exist, I am more inclined to attribute them to the depleting means often too liberally employed, to the continued action of malaria, and to the repetition of paroxysms of fever which should have been checked at once. If epidemics show a strong tendency to fasten upon some particular organ, physicians evince an equally strong one, to follow their example. Since the days of Abernethy and Philip until lately, the liver has nearly filled up the pathological anatomy of some physicians of our country, and calomel their therapeutics. So often have I seen patients drugged for bilious and liver affections where none existed; and so very rarely have I seen unequivocally diseased livers in dead bodies, that I look with suspician on those cases so often spoken of under the vague terms of 'hepatic derangements' and 'congested liver.' Nor do I extend to them a greater share of confidence when they are reported in connection with the use of quinine, and asserted to exist as a pretty constant cause, or part, of dysentery. These notions are so much opposed to my own observations, and savor so strongly of the theories prevalent some fifteen or twenty years ago, that I cannot but receive them with many grains of allowance."

With regard to the *modus operandi* of quinine, the evidence of Dr. B. is necessarily of a negative character. Though classed among "tonics" by writers on the materia medica, the idea that its action depends upon tonic or stimulating properties, is a peculiarly unhappy one, for it has prevented its use, no doubt, in many a case of malarial fever which it would have cured with certainty; fever, it is reasoned, is a sthenic condition of the system, and a tonic or stimulant would certainly be inadmissible in it, according to the rules of art. The terms tonic, stimulant, or sedative, convey no just impression of the effects of quinine; its whole force is expended upon the nervous system, and its influence over the circulation is doubtful and uncertain. The effects perceived are those of an anti-periodic, or in more comprehensive phrase, a direct *antidote to the malarial poison.* How this effect is produced, is a question which must remain unanswered. The ultimate action of medicines, like most ultimate causes, thus far lies buried in obscurity, and it is better for us to be aware of our ignorance of the *modus agendi* of quinine, and to be content to use it for the well-known effects which experience has taught us it will produce, than to call it a tonic, stimulant or sedative, terms which involve a false theory, and must lead to erroneous practice.—*Boston Med. and Surg. Journal.*

*Surgeon-General's Office, June,* 1845.

